# Novel sources of drought tolerance in sorghum landraces revealed *via* the analyses of genotype-by-environment interactions

**DOI:** 10.3389/fpls.2022.1062984

**Published:** 2022-12-07

**Authors:** Muluken Enyew, Anders S. Carlsson, Mulatu Geleta, Kassahun Tesfaye, Cecilia Hammenhag, Amare Seyoum, Tileye Feyissa

**Affiliations:** ^1^ Institute of Biotechnology, Addis Ababa University, Addis Ababa, Ethiopia; ^2^ Department of Plant Breeding, Swedish University of Agricultural Sciences, Alnarp, Sweden; ^3^ Ethiopian Biotechnology Institute, Addis Ababa, Ethiopia; ^4^ National Sorghum Research Program, Crop Research Department, Melkassa Agricultural Research Center, Ethiopian Institute of Agricultural Research, Adama, Ethiopia

**Keywords:** abiotic stress, AMMI biplot, chlorophyll content, drought, GGE biplot, sorghum, stay-green

## Abstract

Globally, sorghum is the fifth most important crop, which is used for food, feed and fuel. However, its production and productivity are severely limited by various stresses, including drought. Hence, this study aimed to determine the responses of different drought-tolerance related traits in the Ethiopian sorghum germplasm through multi-environment field trials, thereby identifying novel sources of germplasm that can be used for breeding the crop for drought-tolerance. Three hundred twenty sorghum landraces and four improved varieties were grown at three sites within drought-prone areas (Melkassa, Mieso and Mehoni) in Ethiopia. The targeted traits were chlorophyll content at flowering (CHLF), chlorophyll content at maturity (CHLM), green leaf number at flowering (GLNF), stay-green (SG), flag leaf area (FLA), peduncle length (PDL), and panicle exertion (PAE). Multi-variate analyses of the collected data revealed the presence of high phenotypic variation in all traits. The combined and AMMI Analysis of variance showed that phenotypic variation due to the genotypes was higher for SG, CHLM, CHLF and GLNF and lower for FLA, PE and PDL in comparison with variation due to the environments or genotype by environment interactions. High broad sense heritability was observed for CHLF, CHLM, SG, GLNF, FLA, and PDL, whereas PAE showed moderate heritability. Due to the high heritability of chlorophyll content and the relatively small effect of environmental factors on it, it could serve as a criterion for selecting desirable genotypes for drought-tolerant breeding in sorghum. It has been found that chlorophyll content has a significant positive correlation with stay-green and grain yield, indicating that high chlorophyll content contributes to increasing grain yield by delaying the process of leaf senescence. The analyses of AMMI, GGE biplot, and genotype selection index revealed that several sorghum landraces outperformed the improved varieties with respect to CHLF, CHLM, and SG. Such landraces could serve as novel sources of germplasm for improving drought tolerance through breeding.

## 1 Introduction

Sorghum [*Sorghum bicolor* (L.) Moench] ranks fifth among the most important cereal crops in the world, after maize, rice, wheat, and barley (faostat, 2020). In 2020, global sorghum production was 29.8 million metric tons (MMT) with 1.5 tonnes per hectare (t ha^−1^) of average productivity (Faostat, 2020). It is a food security crop for more than half a billion people in developing countries, mainly in arid and semi-arid regions where moisture stress is a major constraint (Ejeta, 2005). Sorghum is a multipurpose crop, which is used for food, feed, and fuel (Waniska et al., 2016; Stamenković et al., 2020). Due to its gluten-free nature, high starch and protein levels, and high content of condensed health-beneficial compounds, its grain has high nutritional value (Waniska et al., 2016; Dykes, 2019).

Despite its importance, the productivity of sorghum is highly limited by many factors but drought remains the major abiotic constraint. About 80% of sorghum production in the world is under dryland conditions (Assefa et al., 2010) and in Sub-Saharan Africa, sorghum is mainly cultivated in drought-prone areas that cover nearly 60% of the total area (Hadebe et al., 2017). In Ethiopia, 66% of the areas where sorghum is predominantly cultivated are prone to frequent drought (Geremew et al., 2004). Several studies have reported the impact of drought stress on sorghum. Drought stress affects the growth and development of sorghum, which ultimately leads to a substantial reduction in grain yield (Bobade et al., 2019; Queiroz et al., 2019; Abreha et al., 2022). For instance, Assefa et al. (2010) reported that drought stress at the vegetative and reproductive stages reduced the sorghum yield by more than 36% and 55%, respectively.

The northeastern part of Africa, most likely modern Ethiopia and Sudan, is considered to be the center of origin and domestication of cultivated sorghum (Doggett, 1970; Dillon et al., 2007). The presence of high genetic diversity in the Ethiopian sorghum gene pool has been reported by several authors (Adugna et al., 2013; Adugna, 2014; Enyew et al., 2022). The original sources of the most widely used stay-green genotypes B35 and SC56 are Ethiopia and Sudan, respectively. The likelihood of the diverse sorghum gene pool that exists in this region being a potential source of more novel drought tolerance and stay-green genotypes is high. In line with this, Disasa et al. (2016) reported the presence of high genetic diversity in the Ethiopian sweet sorghum germplasm that has not yet been utilized in sorghum breeding programs. Crop landraces and wild relatives are potential sources of desirable genes for crop improvement (Kyratzis et al., 2019; Ochieng et al., 2020). In this regard, the Ethiopian sorghum gene pool could serve as a potential source of resistant/tolerant genotypes against biotic and abiotic stresses.

Multiple drought-tolerant genotypes, such as 00MN7645, QL41, B35, BTx642, SC-56, and E-36-1 have been identified and used in sorghum breeding programs (Tao et al., 2000; Xu et al., 2000; Kebede et al., 2001; Haussmann et al., 2002; Sukumaran et al., 2016). However, it is still necessary to find additional sources of drought and stay-green genotypes to avoid dependence on only a few sources, which is the case in the current sorghum breeding programs across the world. Drought tolerance is a complex trait, controlled by many genes. In addition, various environmental factors affect drought severity, making sorghum breeding for drought tolerance challenging (Tuinstra et al., 1996; Abreha et al., 2022). Nevertheless, selecting drought-tolerant sorghum genotypes is crucial for improving the production of sorghum through the application of modern breeding methods.

Selecting sorghum landraces that have drought tolerance-related traits such as stay-green and high chlorophyll content is a crucial step in sorghum breeding programs. This is because improving such traits, which have moderate to high heritability (Harris et al., 2007; Mutava et al., 2011; Ochieng et al., 2020) could lead to an increased level of drought tolerance of the crop (Harris et al., 2007; Mutava et al., 2011). Drought stress often causes a reduction in chlorophyll content of sorghum plants and promote leaf senescence (Thomas and Ougham, 2014; Hou et al., 2021), which results in reduced grain yields (Borrell et al., 2000; Djanaguiraman et al., 2020). High chlorophyll content has also been associated with improved stay-green in sorghum and reduces post-flowering drought-induced senescence (Harris et al., 2007). Similarly, the growth of panicle exertion and peduncle length were lower in the sorghum plants grown under stress conditions as compared to the control (Maluk, 2018). Furthermore, the panicle exertion was reported to be affected by water stress occurring after floral initiation in sorghum and rice (Tsuda, 1986; Praba et al., 2009). The panicle exertion was also reported to increase harvest index and improve yield in wheat (Liang et al., 2013). Therefore, the use of drought related traits such as stay-green, chlorophyll content, number of leaf per plant, leaf area, panicle exertion and peduncle length as selection criteria to identify drought tolerant genotypes could lead to increased grain yield in sorghum in drought prone areas. However, not much known about the genotype by environment interactions and the stability of drought tolerance related traits in sorghum

The analyses of genotype stability across different environments have been carried out by using different statistical models as reviewed by Pour-Aboughadareh et al. (2022). Additive main effects and multiplicative interaction (AMMI) and genotype main effects plus genotype by environment interaction (GGE) biplot models are the most effective and commonly used models for the analyses of stability, adaptability and ranking of genotypes and for selecting suitable mega environments. In AMMI, genotype type stability is determined through the analysis of AMMI stability value (ASV) (Purchase et al., 2000), sums of the absolute value of the IPCA scores (SIPC), averages of the squared eigenvector values (EV) (Sneller et al., 1997), weighted average of absolute scores (WAAS) (Olivoto et al., 2019), and absolute value of the relative contribution of IPCAs to the interaction (ZA) (Zali et al., 2012). Low values of these parameters suggest high stability of the genotypes across environments. Most sorghum production areas in Ethiopia are in arid or semi-arid regions with high rainfall variability and low soil water storage. Consequently, the total annual rainfall and its distribution throughout the sorghum-growing season play a critical role in sorghum grain yield. The availability of sufficient moisture in the soil at certain growth stages is particularly crucial for obtaining a good grain yield (Assefa et al., 2010). Evaluation of moisture availability during the growing season of sorghum (and other crops) in Ethiopia for a long period led to Melkassa, Mehoni, and Mieso being designated moisture stress sites (dry lowland) suitable for testing sorghum’s tolerance to moisture stress. Even though these sites receive annual rainfall that may appear to be sufficient for growing crops without much moisture stress, they are considered moisture-stressed due to factors such as inefficient distribution of rain during the crop-growing season, a relatively low moisture-holding capacity of the soils, and occasional high temperatures. Accordingly, these sites have been used as suitable testing sites for moisture stress-related research on sorghum (Wagaw et al., 2020; Wondimu et al., 2020; Teressa et al., 2021) including for the present study.

The objectives of the present study were to determine the drought stress response and stability of the Ethiopian sorghum landraces through the determination of genotype by environment interactions of different traits. This was done through the application of AMMI and GGE biplot models to identify novel sources of drought tolerance for use in sorghum breeding programs.

## 2 Materials and methods

### 2.1 Plant materials

A total of 324 sorghum genotypes representing 320 landrace accessions and four improved varieties were used for the field experiment (**Supplementary Table 1**). Among the 320 landrace accessions, 261 were selected from sorghum landrace accessions supplied by Ethiopian Biodiversity Institute (EBI) to Melkassa Agricultural Research Center (MARC), while 59 accessions were collected for this study from farmers’ fields in drought-prone areas. Among the improved varieties, Argiti, ESH4 and Melkam are drought tolerant and high yielding genotypes, whereas B35 is a stay-green but low yielding genotype.

### 2.2 Field experiment sites, layout and management

The 324 sorghum genotypes were evaluated during the main crop-growing season in 2019 at three commonly used testing sites for moisture-stress research: Melkassa (MK), Mehoni (MH), and Mieso (MS). The sites are within moisture stress areas in Ethiopia where smallholder farmers predominantly grow sorghum. The detail descriptions of the sites are presented in **Supplementary Table 2**. The experimental layout was alpha lattice design with two replications at each site. The plot size was 2.25 m^2^ (3 m × 0.75 m). On each plot, the seeds were sown in a single 3 m long row and planting was done manually followed by thinning to 0.20 m space between plants. The recommended amount of 100 kg ha^-1^ DAP fertilizer was applied during planting and 50 kg ha^-1^ of urea was side dressed 40 days after planting. The recommended field management practices were followed for sorghum field experiments.

### 2.3 Phenotypic data collection

All phenotypic data were collected from five randomly selected and tagged plants in each plot. Chlorophyll content of the flag leaves of sorghum plants was measured at flowering (CHLF) and maturity (CHLM) stages using SPAD chlorophyll meter. Green leaf number was recorded by counting the number of green leaves of each plant at a flowering stage (GLNF). Based on Stickler et al. (1961) approach, flag leaf area was calculated as the product of maximum flag leaf length, maximum flag leaf width, and 0.75. Stay-green (SG) was scored at maturity based on visual ratings (Wanous et al., 1991; Reddy et al., 2007) using 1 to 5 scale (1 = > 75% dried leaves and 5 = 0 to 10% dried leaves). Peduncle length (PDL) was measured from the base of the first node where the sheath of the flag leaf is attached to the bottom of the panicle, while panicle exertion (PAE) was measured from the sheath of the flag leaf to the base of the panicle. In addition, the grain yield data published in (Enyew et al., 2021) was used to determine its correlation with the traits targeted in this study.

### 2.4 Data analysis

The combined analysis of variance (ANOVA) was carried out using the mixed linear model in R software (Team, R.C., 2020). The genotype, environment and G×E interaction effects on the total phenotypic variance of each trait were then determined. The G×E interaction effect, genotype adaptability and stability across the three environments were determined using the AMMI model in GENSTAT software (International, V. 2009). The AMMI stability parameters, ASV, SIPC, EV, WAAS and ZA were estimated to rank the genotypes according to their phenotypic stability through the application of metan and Agricola package in the R software (de Mendiburu and de Mendiburu, 2019; Olivoto and Lúcio, 2020). To identify top performing and stable genotypes, genotype selection index (GSI) of each genotype was calculated by adding ASV and genotype mean ranking, as described in Purchase et al. (2000), with R software. The GGE biplot analysis was also performed using GENSTAT software (International, V. 2009). The cor() function in R was used to determine the correlation between each pair of traits using the Pearson’s method (Peterson et al., 2014). The level of significance for each correlation was determined *via* the application of cor.test() function in R software. The scatter plots and histograms were generated by applying the chart.Correlation() function within the Performance Analytics package (Peterson et al., 2014). The broad-sense heritability (H^2^) of all traits was calculated by META-R software (Alvarado et al., 2020).

## 3 Results

### 3.1 Phenotypic trait variation and heritability

**Figure 1:**
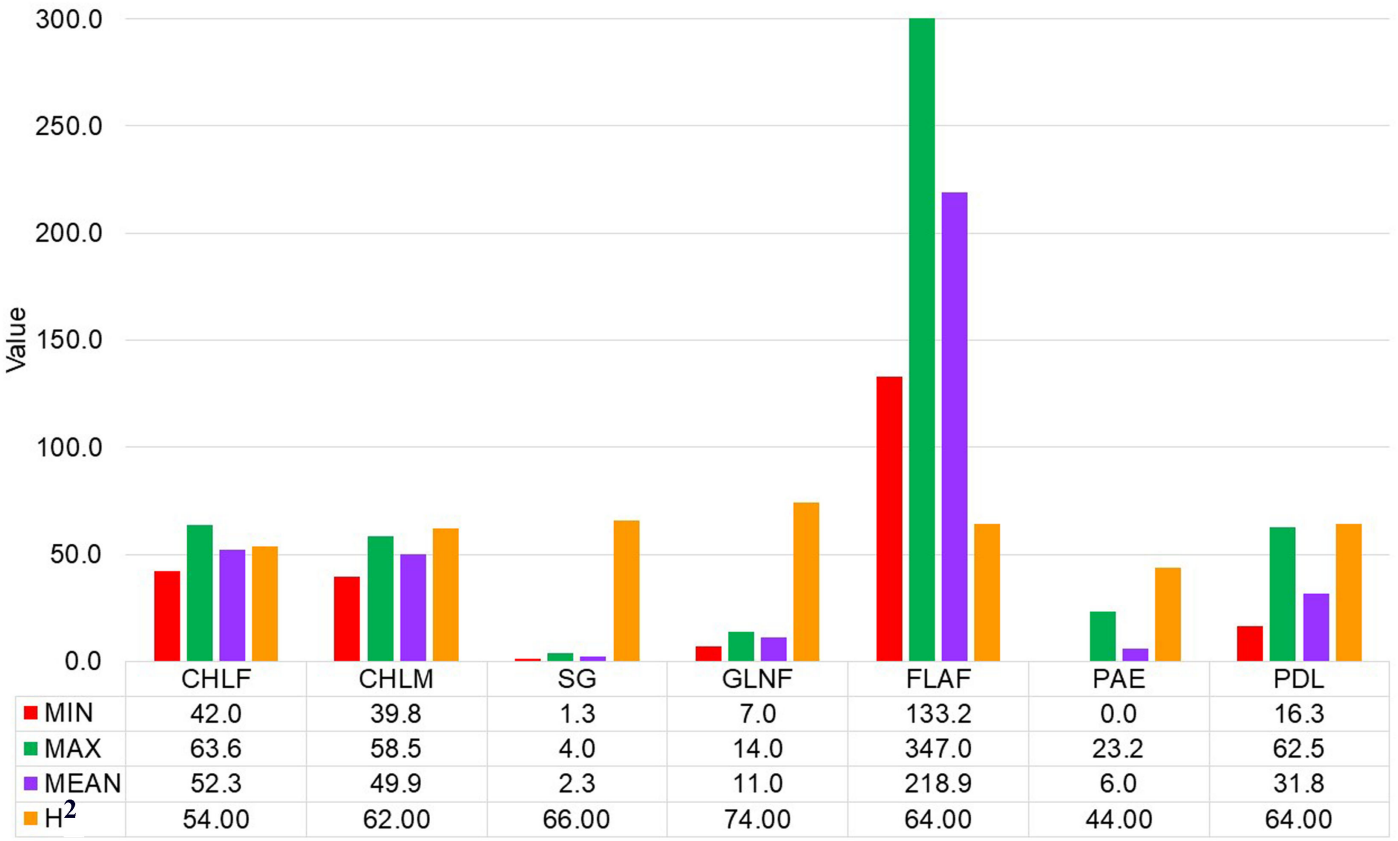
Summary statistics and heritability of chlorophyll content at flowering (CHLF, SPAD value), chlorophyll content at maturity (CHLM, SPAD value), stay-green (SG, visual rating from 1 to 5 scale), green leaf number at a flowering (GLNF), flag leaf area in square centimeter (FLA), panicle exertion in centimeter (PE) and peduncle length in centimeter (PDL).

**Figure 2:**
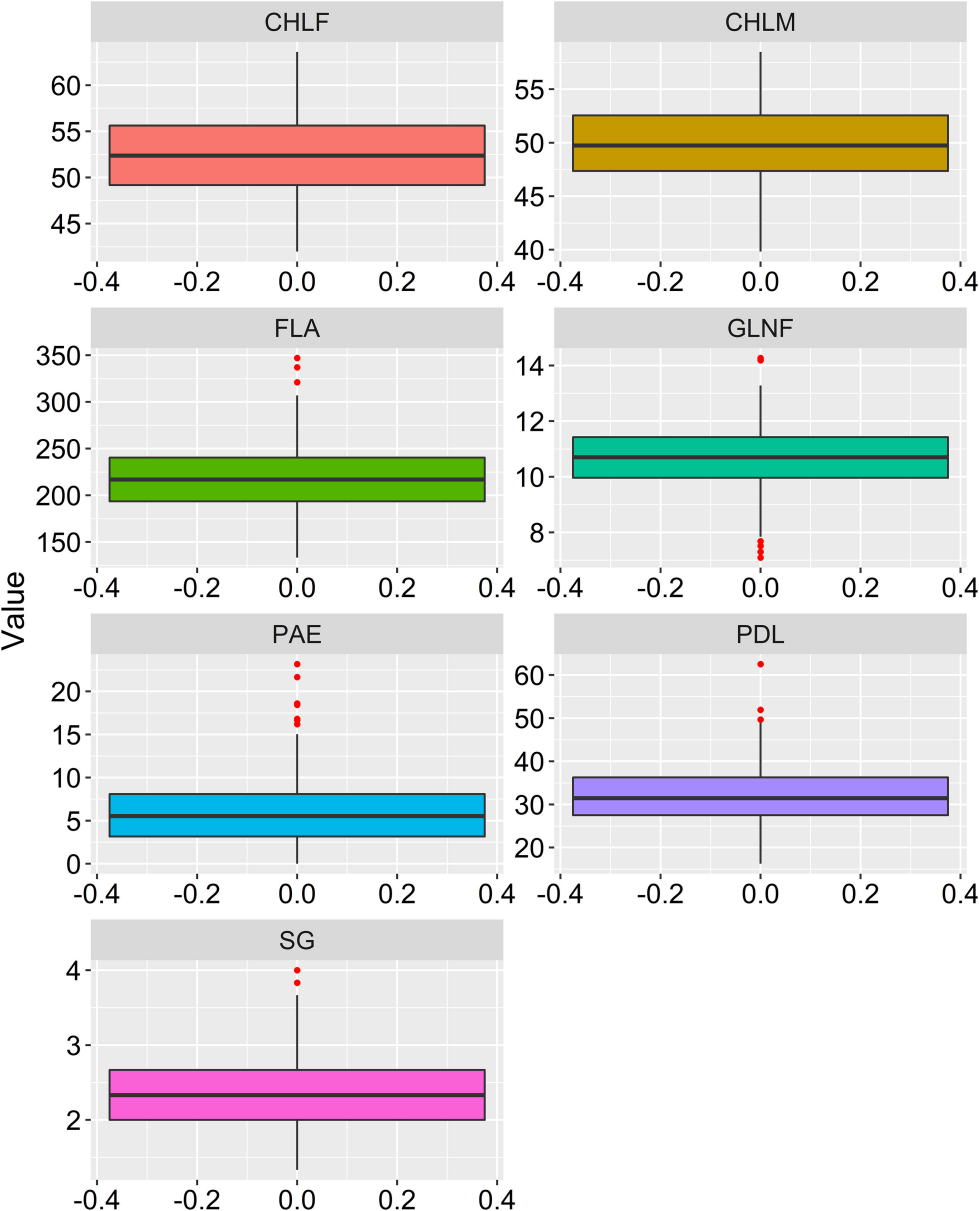
Box-plot showing phenotypic distribution of the drought related traits. CHLF, Chlorophyll content at flowering (CHLF, SPAD value), chlorophyll content at maturity (CHLM, SPAD value), stay-green (SG, visual rating from 1 to 5 scale), green leaf number at a flowering (GLNF), flag leaf area in square centimeter (FLA), panicle exertion in centimeter (PE) and peduncle length (PDL) in centimeter.

**Figure 3:**
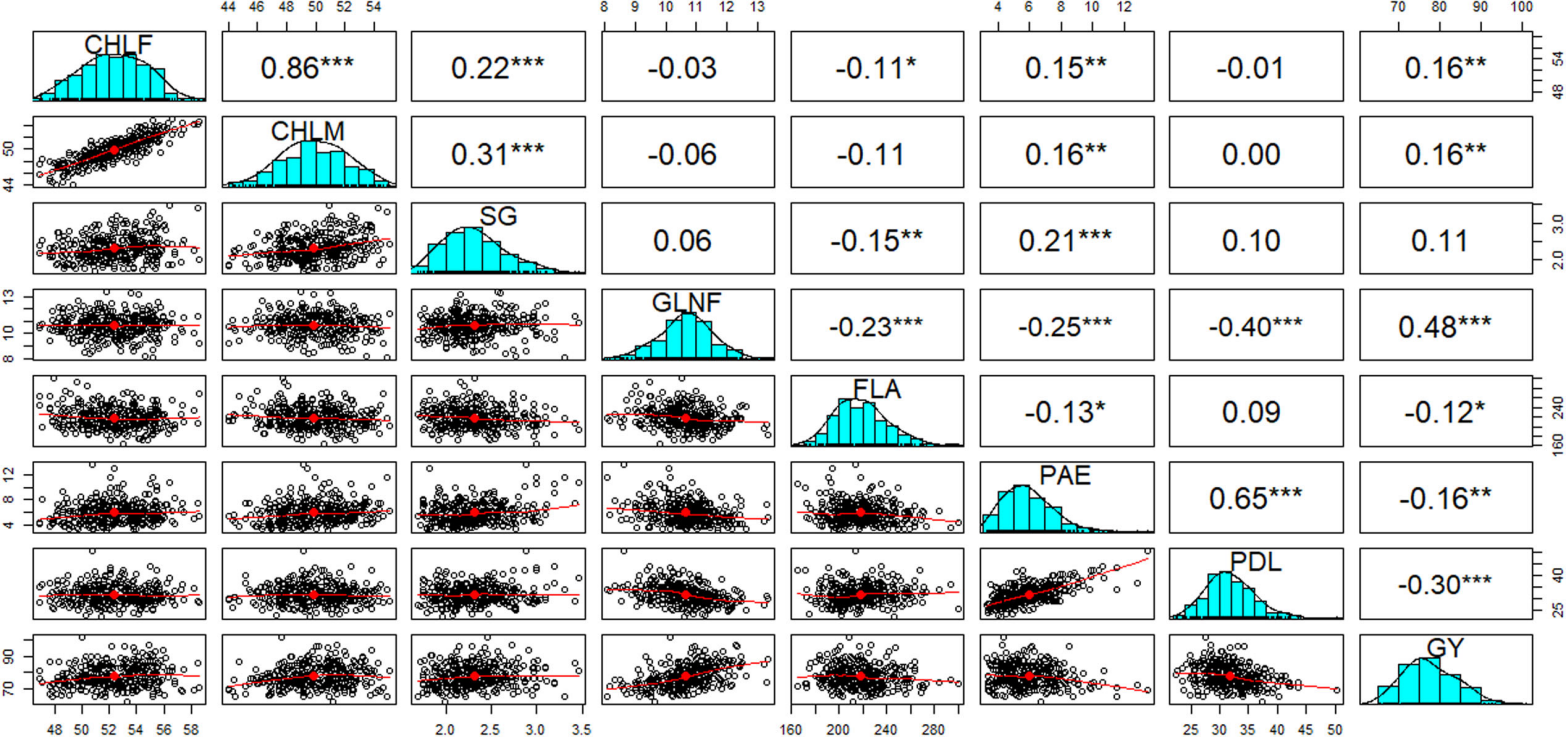
Pearson correlation coefficients (above diagonal) between eight drought-tolerance related traits and their corresponding scatter plots of measured individuals of the 324 genotypes (below diagonal). Histograms for chlorophyll contents at flowering (CHLF) and maturity (CHLM), stay green (SG), green leaf number at flowering (GLNF), flag leaf area (FLA), panicle exertion (PAE), peduncle length (PDL), and grain yield (GY) are displayed along the diagonal. The red lines through the scatter plots represent the line of best fit. *, **, and *** indicate that the correlations were significant at 0.05, 0.01 and 0.001 levels of significance, respectively. Correlation values without asterisk were not significant.

Large variations within drought-tolerance related traits were recorded in the sorghum genotypes used in the present study at three drought-prone sites in Ethiopia (**Table 1** and **Figure 1**). A normal frequency distribution was observed for all traits for combined environments as showed in box plot and histogram (**Figures 2**, **3**). The chlorophyll content of the genotypes at a flowering stage (CHLF) varied from 42.0 to 63.6 with a mean SPAD value of 52.3. Whereas the chlorophyll content of the genotypes at maturity (CHLM) varied from 39.8 to 58.5 with a mean SPAD value of 49.9. Stay-green (SG) values varied from 1.3 to 4.0 with a mean value of 2.3 while the average number of green leaves at flowering stage (GLNF) was 11.0 with individual values ranging from 7.1 to 14.0. Flag leaf area (FLA) varied from 133.2 to 347.0 cm^2^ with a mean value of 218.9 cm^2^. The mean values of panicle exertion (PE) and peduncle length (PDL) were 6.0 cm and 31.8 cm with individual values ranging from 0.0 to 23.2 cm and 16.3 to 62.5 cm, respectively (**Figure 1**). The broad sense heritability ranged from 44% for PAE to 74% for GLNF. High broad sense heritability was observed for CHLF, CHLM, SG, GLNF, FLA, and PDL whereas PAE showed moderate level of heritability (**Figure 1**).

**Table 1:** AMMI analysis of variance for seven drought-tolerance related traits of 324-sorghum genotype across three environments.

Source	Trait	CHLF		CHLM		SG		GLNF		FLA		PAE		PDL	
		DF	MS	%TV	MS	%TV	MS	%TV	MS	%TV	MS	%TV	MS	%TV	MS	%TV
**Total**	1943	39.4		39.4		0.88***		3.385		5836		47.7		121	
**Trt**	971	52.3***		52.2***		1.16***		4.619***		9399***		64***		160.8***	
**Block**	3	175.4***		83.5***		9.21***		2.234		6456**		136.5**		5784.6***	
**GEN**	323	83.9***	35.6	1405.1***	35.5	1.66***	32.1	8.92***	43.8	7491***	21.4	85.7***	30.0	269.9***	11.4
**ENV**	2	1398.7***	3.7	176.3***	3.7	113.07***	13.5	21.038***	0.6	2471073***	43.7	1804.8***	3.9	1926.8	81.6
**G**×**E**	646	32.3**	27.4	32.4**	27.5	0.56***	21.4	2.418	23.8	2731**	15.6	47.8***	33.4	100.8***	4.3
**IPCA1**	324	41***	63.7	41.2**	72.7	1.0***	89.9	3.506***	72.7	3464***	63.6	58.9***	61.9	132.9***	66.1
**IPCA2**	322	23.5	36.3	23.5	27.3	0.12	10.1	1.323	27.3	1994	36.4	36.6**	38.1	68.5	33.9
**Error**	969	26.1	33.3	26.1	33.3	0.57	33.1	2.153	31.7	2264	19.4	31.1	32.7	63.6	2.7

***, **, * = Significant at 0.001, 0.01 0.05 levels of significance, respectively; DF, Degrees of freedom; Trt, Treatment; GEN, Genotype; ENV, Environment; G×E, Genotype by environment interaction; IPCA, Interaction principal component axis; MS ,Mean square; %TV, Percentage of total variance explained; CHLF, Chlorophyll content at flowering; CHLM, Chlorophyll content at maturity; SG, Stay-green; GLNF, Green leaf number at flowering; FLA, Flag leaf area; PAE, Panicle exertion; PDL, Peduncle length; Source, Source of variation.

The analysis of average genotype performance indicated that several genotypes outperformed the drought tolerant and high-yielding varieties (Melkam, Argiti, and ESH4) as well as the stay-green genotype (B35) in terms of chlorophyll content at the flowering stage (**Table 2**). The top three genotypes were G48, G65, and G66 while the bottom three were G10, G254 and G307 for this trait (**Table 2**). Similarly, several genotypes outperformed Melkam, Argiti, and ESH4 with respect to their chlorophyll content at maturity and stay-green traits. However, only G59 for CHLM and G22 and G66 for SG had higher mean values than B35 (**Table 2**). The landrace accessions that had better mean performance than the improved varieties with respect to their CHLF, CHLM, and SG were considered as potential novel sources of functional drought tolerance. Highest number of leaves were recorded by genotype G163, G202 and G242 whereas genotype G266, G268 and G289 showed highest flag leaf area. With regard to PAE, the highest mean was recorded by genotype G41 which was followed by G118, G146 and G152. Genotype G41, G118 and G152 showed the highest mean value of PDL (**Table 2**).

**Table 2:** Average values of the top 10, bottom 5 sorghum landrace and known drought tolerant and high yielding varieties (Melkam, Argiti, and ESH4) as well as the stay-green genotype (B35).

Trait	CHLF		CHLM			SG			GLNF			FLA			PAE			PDL		
Top ten Genotype																				
NO	Gen	Mean ± SE		Gen	Mean ± SE		Gen	Mean ± SE		Gen	Mean ± SE		Gen	Mean ± SE		Gen	Mean ± SE		Gen	Mean ± SE
1	G65	63.6 ± 4.9		G59	58.5 ± 3.4		G22	4.0 ± 0.4		G163	14 ± 0.5		G266	347.0 ± 24.4		G41	23.2 ± 5.1		G41	62.5 ± 7.9
2	G66	63.4 ± 3.9		G316	57.6 ± 4.0		G66	4.0 ± 0.3		G202	14 ± 0.5		G268	337.0 ± 46.1		G146	21.7 ± 4.2		G118	51.9 ± 3.9
3	G48	63.4 ± 2.4		G65	57.5 ± 2.9		G118	3.7 ± 0.3		G242	14 ± 0.4		G289	307.0 ± 25.5		G152	18.4 ± 3.5		G152	49.7 ± 2.4
4	G175	62.3 ± 2.1		G114	57.5 ± 1.7		G170	3.7 ± 0.6		G302	13 ± 0.7		G12	303.7 ± 26.0		G118	16.8 ± 2.6		G149	48.9 ± 3.9
5	G316	62.1 ± 3.9		G48	57.1 ± 3.4		G53	3.5 ± 0.4		G200	13 ± 0.5		G271	302.6 ± 46.6		G22	16.7 ± 2.6		G106	48.5 ± 4.2
6	G52	61.4 ± 3.1		G18	56.9 ± 1.8		G97	3.5 ± 0.4		G193	13 ± 0.4		G287	299.7 ± 53.5		G106	16.2 ± 3.0		G23	48.1 ± 2.3
7	G160	61.0 ± 3.0		G78	56.9 ± 2.6		G274	3.5 ± 0.4		G93	13 ± 0.4		G65	298.6 ± 33.0		G42	15.0 ± 4.6		G169	47.3 ± 8.4
8	G36	60.9 ± 3.0		G185	56.8 ± 1.6		G291	3.5 ± 0.4		G203	13 ± 0.4		G34	297.3 ± 19.6		G79	15.0 ± 5.9		G79	46.7 ± 8.0
9	G40	60.6 ± 2.6		G43	56.6 ± 1.6		G43	3.3 ± 0.5		G97	13 ± 0.5		G149	295.9 ± 50.9		G129	14.8 ± 2.5		G248	46.7 ± 2.6
10	G59	60.6 ± 4.2		G66	56.6 ± 3.4		G83	3.3 ± 0.2		G239	13 ± 0.5		G30	290.2 ± 43.2		G169	14.8 ± 5.6		G263	46.6 ± 3.3
**Bottom five Genotype**																				
1	G26	43.4 ± 4.4		G167	41.4 ± 2.0		G300	1.5 ± 0.2		G17	8.0 ± 0.4		G178	151.0 ± 16.7		G245	0.0 ± 0.0		G315	19.2 ± 2.6
2	G318	43.0 ± 2.8		G27	41.0 ± 2.1		G304	1.5 ± 0.2		G205	8.0 ± 1.1		G37	145.8 ± 13.7		G260	0.0 ± 0.0		G178	18.9 ± 3.9
3	G307	42.4 ± 3.9		G8	40.7 ± 2.3		G44	1.3 ± 0.2		G41	8.0 ± 0.9		G70	145.6 ± 10.8		G294	0.0 ± 0.0		G300	18.8 ± 1.8
4	G254	42.2 ± 3.0		G293	40.5 ± 1.1		G188	1.3 ± 0.2		G208	8.0 ± 0.5		G169	143.9 ± 10.2		G300	0.0 ± 0.0		G317	17.0 ± 2.2
5	G10	42.0 ± 3.6		G7	39.8 ± 2.9		G265	1.3 ± 0.3		G113	8.0 ± 0.6		G106	133.2 ± 13.1		G302	0.0 ± 0.0		G295	16.3 ± 2.3
**Four improved varieties**																				
1	G321	58.2 ± 3.9		G321	58.4 ± 3.0		G321	3.8 ± 0.4		G323	8.4 ± 0.8		G323	321.0 ± 46.9		G322	3.5 ± 2.3		G322	27.0 ± 4.9
2	G324	56.3 ± 1.5		G324	55.3 ± 1.8		G324	3.0 ± 0.3		G322	7.8 ± 0.5		G322	221.3 ± 24.9		G324	5.3 ± 3.3		G324	33.7 ± 6.3
3	G322	54.5 ± 1.0		G322	53.4 ± 1.6		G322	2.5 ± 0.2		G324	7.4 ± 0.5		G321	218.5 ± 26.5		G323	6.5 ± 2.9		G323	34.4 ± 4.0
4	G323	48.3 ± 2.9		G323	46.8 ± 1.9		G323	2.2 ± 0.3		G321	7.0 ± 1.0		G324	205.9 ± 32.3		G321	18.6 ± 5.3		G321	35.3 ± 5.2

Chlorophyll content at flowering (CHLF, SPAD value), chlorophyll content at maturity (CHLM, SPAD value), stay-green (SG, visual rating from 1 to 5 scale), green leaf number at a flowering (GLNF), flag leaf area in square centimeter (FLA), panicle exertion in centimeter (PE) and peduncle length in centimeter (PDL). Gen, Genotype; SE, standard error; G321, B35; G322, Melkam; G323, Argiti; G324, ESH4.

### 3.2 Correlation between traits

Significant positive and negative correlations were observed between the traits studied (**Figure 3**). The highest positive correlation was observed between CHLF and CHLM (r = 0.86; *P < 0.001*). These two traits showed highly significant positive correlations with SG (*P < 0.001*). On the other hand, GLNF was negatively correlated (*P < 0.001*) with FLA (r = -0.23), PAE (r = -0.25) and PDL (r = -0.40). GY showed significant positive correlations with CHLF (r = 0.16), CHLM (r = 0.16), and GLNF (r = 0.48) and significant negative correlations with FLA (r = -0.12), PAE (r = -016), and PDL (-0.30). PAE showed a highly significant positive correlation with PDL (r = 0.65; *P < 0.001*). PAE also showed significant positive correlation with CHLF (r = 0.15), CHLM (r = 0.16), and SG (r = 0.21) (**Figure 3**).

### 3.3 AMMI analysis of variance

The result of combined and AMMI analysis of variance revealed that the genotype, environment, and G×E interaction effects were highly significant (*P < 0.001*) for all traits studied (**Table 1** and **Supplementary Table 3**). The Analysis of variance showed that the phenotypic variation due to genotype was higher than the variation due to environment and G×E interaction for SG, CHLM, CHLF, and GLNF. However, it was lower than the variation due to the environment for FLA and PDL, and due to G×E interaction for PAE. For SG, CHLM, CHLF and GLNF, the genotypic variance explained 32.1%, 35.5%, 35.6%, and 43.8% of the total phenotypic variance whereas the environmental variance accounted for 3.7%, 13.5%, 3.7%, 3.7%, and 0.6% of the total phenotypic variance, respectively (**Table 1** **)**. In the case of FLA, the environmental variance accounted for 43.7% of the total variance whereas genotype and G×E interaction contributed 21.4% and 15.6% to the total variance, respectively. The proportion of the total variance explained by genotype, environment, and G×E interaction for PAE was 30.0%, 3.9%, and 33.4%, respectively. For PDL, environment effects accounted for 81.6% of the total variance whereas genotype and G×E interaction contributed 11.4% and 4.3% to the total variance, respectively (**Table 1**).

### 3.4 AMMI stability analysis

The AMMI stability analysis revealed that the top three highly stable genotypes with low G×E interaction for CHLF were G244, G222, and G201, as their AMMI stability values (ASV) of 0.09, 0.10, and 0.11 were the lowest (**Supplementary Table 4**). These genotypes were also the top three stable genotypes with low EV values for CHLF. Whereas G244, G201 and G197 were the top three stable genotypes according to their SIPC values. Genotypes G244, G222, and G109 had the smallest WAAS and ZA values, and therefore, they were the most stable for CHLF according to these two parameters (**Supplementary Table 4**). For chlorophyll content at maturity (CHLM), G81, G53, and G255 were the top three most stable genotypes with ASV of 0.02, 0.07, and 0.08, respectively. According to EV, SIPC, WAAS and ZA values, G114, G302, and G240 were the most stable genotypes across the environments (**Supplementary Table 4**). However, not all highly stable genotypes for CHLF and CHLM have high chlorophyll content. Hence, in order to determine genotypes that combine high performance and high stability, the genotype selection index (GSI) is considered. Genotypes G222, G53, and G239 were identified as having high mean performance with stability for CHLF based on their GSI values. Similarly, G53, G308, and G54 were identified as having high stability and mean performance for CHLM based on their GSI (**Table 3**), and hence are desirable for use in sorghum breeding programs.

**Table 3:** The mean performance and the stability of the top ten and bottom five sorghum genotypes ranked by the genotype selection index (GSI) for chlorophyll content at flowering (CHLF), chlorophyll content at maturity (CHLM) and stay-green (SG).

Trait	CHLF				CHLM				SG			
Top 10 genotype												
	Gen	Mean ± SE	ASV	GSI	Gen	Mean ± SE	ASV	GSI	Gen	Mean ± SE	ASV	GSI
1	G222	58.7 ± 1.6	0.1	26	G53	54.7 ± 1.0	0.1	40	G66	4.0 ± 0.3	0.4	28.5
2	G53	59.2 ± 1.8	0.3	46	G308	54.5 ± 2.1	0.2	52	G17	2.8 ± 0.2	0.2	56.5
3	G239	57.1 ± 1.1	0.1	64	G54	54.8 ± 1.5	0.2	56	G97	3.5 ± 0.4	0.4	64.5
4	G320	58.4 ± 1.9	0.3	71	G28	54.4 ± 1.3	0.2	59	G72	3.0 ± 0.3	0.4	77
5	G58	57.8 ± 1.8	0.3	72	G58	54.3 ± 1.4	0.2	62	G239	3.0 ± 0.4	0.4	78
6	G74	59.0 ± 2.9	0.4	73	G140	53.8 ± 1.1	0.2	65	G308	3.0 ± 0.3	0.4	86.5
7	G185	56.7 ± 1.8	0.2	81	G18	56.9 ± 1.8	0.4	74	G249	3.0 ± 0.4	0.4	103
8	G62	56.9 ± 1.5	0.3	93	G259	53.3 ± 1.6	0.2	75	G115	3.0 ± 0.4	0.4	113
9	G90	57.7 ± 1.9	0.4	94	G232	54.4 ± 2.4	0.3	76	G77	3.2 ± 0.3	0.5	126
10	G232	58.3 ± 5	0.4	95	G324	55.3 ± 1.8	0.4	85	G316	2.5 ± 0.2	0.4	129
**Bottom five genotype**												
1	G307	42.4 ± 3.9	1.5	613	G7	39.8 ± 2.9	1.4	596	G35	1.5 ± 0.3	1.1	484
2	G26	43.4 ± 4.4	1.8	622	G303	45.5 ± 3.9	2.8	609	G45	1.5 ± 0.3	1.1	463
3	G272	43.8 ± 4.0	1.9	624	G282	44.4 ± 4.6	1.8	613	G188	1.3 ± 0.4	1.7	565
4	G166	44.2 ± 4.7	2.2	635	G5	43.6 ± 3.3	1.7	616	G265	1.3 ± 0.5	1.7	567
5	G10	42.0 ± 3.6	1.9	635	G10	43.0 ± 2.7	2.0	630	G44	1.3 ± 0.4	1.7	566

Gen , genotype; ASV, AMMI stability value; GSI, genotype selection index; SE, standard error. Low GSI values indicate high mean performance and stability of genotypes.

### 3.5 GGE biplot analysis

#### 3.5.1 Which-Won-Where polygon view of GGE biplot

**Figure 4:**
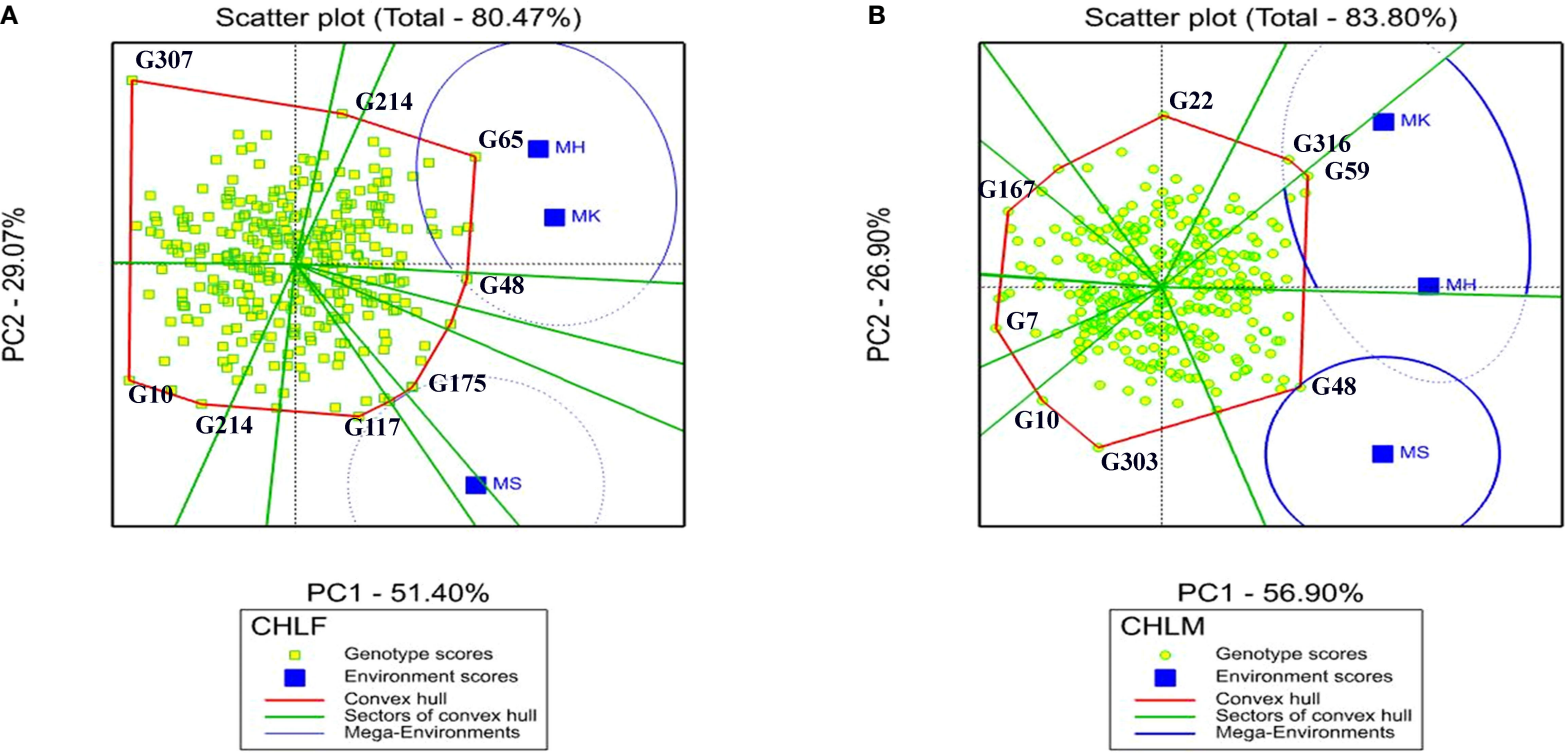
The GGE biplot Which-Won-Where polygon view of the 324 sorghum genotypes for **(A)** chlorophyll content at flowering (CHLF) and **(B)** chlorophyll content at maturity (CHLM), revealing the top performing sorghum genotypes within each environment and mega environments (MGEs). The genotypes located at the vertices of the polygons are the top-performing genotypes in each mega environment for the corresponding trait. MH, Mehoni; MK, Melkassa; MS, Mieso.

The interaction pattern between genotypes and environments and the best performing genotypes were visualized through the Which-Won-Where polygon view of the GGE biplot (**Figure 4**). The polygon in GGE biplot was portrayed by connecting the vertex genotypes, which were located far from the biplot origin, with red straight lines and all the other genotypes were enclosed within the polygon. For CHLF, the vertex genotypes were G65, G48, G175, G117 G214, G10, and G307 while G116, G59, G48, G303, G10, G7, G167, and G22 were the vertex genotypes for CHLM (**Figure 4**). Therefore, these two sets of genotypes were the most responsive to environmental interactions for CHLF and CHLM in that order.

In “which-won-where” GGE biplot, lines from the origin divide the biplot into different sectors and create different mega environments (MGEs) (Yan and Tinker, 2005; Yan and Tinker, 2006). In this study, two MGEs were revealed for both CHLF and CHLM. Environments MK and MH jointly formed an MGE for both CHLF and CHLM while MS falls under a separate MGE (**Figure 4**). Inside the sector containing the first mega environment for chlorophyll content at flowering, genotypes at the vertices of the polygon were G65 and G48 in the environment MK and MH, and G175 and G117 in the MS environment. For CHLM, the genotype at the vertices of the polygon was G48 in the environment MS and G316 and G59 in MK and MH environment, indicating that they are the top performer in their respective environments. According to the GGE biplot analysis, the first two PCs accounted for 80.47% and 83.80% of the total variation in G×E interactions for CHLF and CHLM, respectively (**Figure 4**).

#### 3.5.2 Ranking genotypes based on their mean performance and stability

**Figure 5:**
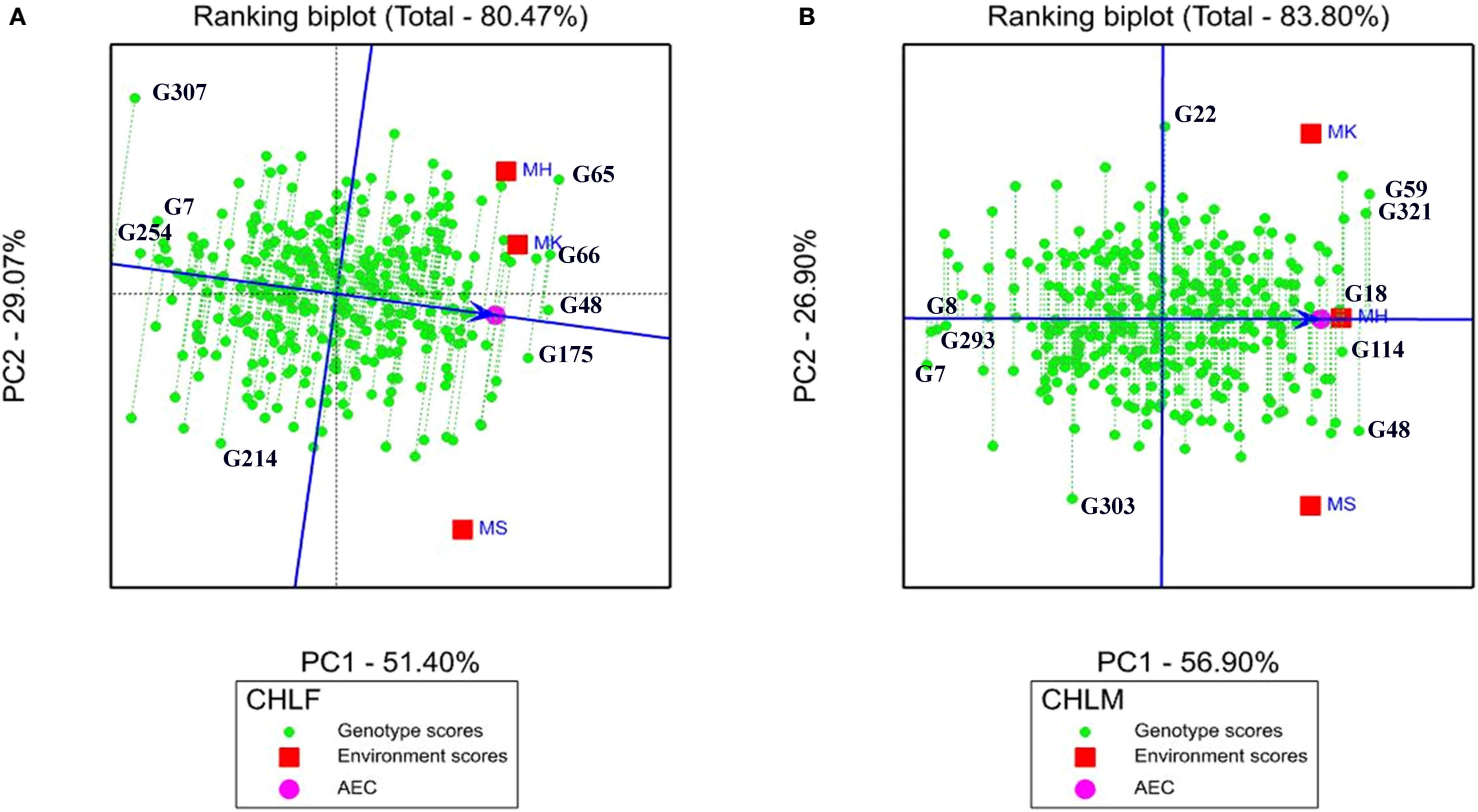
Genotype focus scaling of ranking biplot indicating stability and mean ranking of the 324 sorghum genotypes for **(A)** chlorophyll content at flowering (CHLF) and **(B)** chlorophyll content at maturity (CHLM). The blue arrowhead line that passes through the origin shows genotypes with higher mean performance and the green dotted lines extending from the blue arrowhead line show the level of stability of the genotypes (the longer the dotted line the less stable the genotype is). AEC, average environment coordinate; MH, Mehoni; MK, Melkassa; MS, Mieso.

The rank of mean performance and stability of genotypes were evaluated in ranking biplot through the average environment coordinate (AEC) as described in (Yan, 2001). The average environment axis (AEA) was denoted by a single arrowhead line that passes through the biplot origin indicating a higher mean performance of a genotype in the ranking biplot. In the present study, the ranking biplot AEC revealed that genotypes G48, G66, G65, and G175 had high mean chlorophyll content at the flowering stage whereas genotypes G307, G254, and G7 showed the lowest chlorophyll content (**Figure 5A**). For chlorophyll content at maturity, genotypes G59, G321, G48, G114, and G18 had high mean performance while G7, G293, and G8 showed the lowest mean performance (**Figure 5B**).

In the ranking biplot, the length of the vectors (green dotted lines in the graph) between the genotype positions and the AEA indicates the stability levels of the genotypes (**Figure 5**). The best performing and stable genotypes are those that are located far from the biplot origin but on the AEA or close to it. Hence, G48, G175, and G66 were the most stable genotypes with high mean chlorophyll content at the flowering stage as they are located far from the origin and have shorter vectors from the AEA, whereas G214 and G307 were the least stable genotypes, having the longest vector from the AEA (**Figure 5A**). For the chlorophyll content at the maturity stage, G18 and G114 (**Figure 5B**) were the most stable whereas G22 and G303were the least stable.

## 4 Discussion

This study revealed that there exists substantial phenotypic variation within the Ethiopian sorghum gene pool for the drought-tolerance related traits studied across three locations. The observed phenotypic variation in this study and previous studies on Ethiopian sorghum (Girma et al., 2019; Enyew et al., 2021) suggests that tolerant genotypes to abiotic stresses can be obtained through screening its diverse gene pool. The observed high broad-sense heritability for most of the traits analyzed suggests a major role of genetics in the phenotypic variation of the traits, which could be used for improving drought-tolerance related traits through selection. In line with this study, a high broad-sense heritability for drought-tolerance related traits, such as chlorophyll content and stay-green were reported in previous studies in sorghum (Kapanigowda et al., 2013; Naoura et al., 2019; Ochieng et al., 2020). However, low heritability of chlorophyll content in this crop has also been reported (Badran (2020). This difference may be due to the variation in the genotype, environment and genotype by environment interaction effects.

The significant positive correlation observed among the chlorophyll content and grain yield is in agreement with previous studies in sorghum (Ochieng et al., 2020; Abreha et al., 2022) and other crops (Kamal et al., 2019). Similarly, chlorophyll content showed a highly significant positive correlation with stay-green (SG). This is due to the ability to maintain high chlorophyll content during drought-stress conditions, thereby delaying the process of leaf senescence. Xu et al. (2000) reported the correlation of leaf chlorophyll content and SG in sorghum during post-flowering drought suggesting the use of these traits for screening drought tolerance and yield in sorghum. Sorghum genotypes that maintain high chlorophyll content under drought stress are more likely to produce higher grain yields than non-SG types. This indicates that the stay-green genotypes identified in the current study are highly valuable genetic resources for breeding sorghum for drought tolerance. The highly significant positive correlation observed between GLNF and grain yield in this and previous reports on sorghum (Reddy et al., 2014) indicate that GLNF, being easy to measure, could serve as one of the criteria for selecting genotypes that can be used in sorghum breeding programs that aim at developing drought tolerant cultivars. PAE showed significant positive correlation with CHLF, CHLM and SG. Similarly, Maluk (2018) reported that PAE positive association with stay-green trait. This may be due to the genotypes which have stay-green trait may maintain high leaf water potential which allows the plant to maintain the extension of the PAE and PDL under drought stress conditions.

The present study confirmed that genotype, environment and G×E interaction have significant effects on the drought-tolerance related traits (CHLM, CHLF, SG, GLNF, FLA, PE and PDL). This highlights the significance of multi-environment field trials for genotype evaluations aimed at the identification of stable genotypes with a high mean performance for the studied traits. The phenotypic variation due to genotype was high for SG, CHLM, CHLF and GLNF, indicating that genotypes played a larger role in phenotypic variation than environments. The effects of environment and G × E interaction were higher for FLA, PE and PDL, indicating that the effects of genotypes on the phenotypic variation in these traits were lower.

High genetic variation exists in the Ethiopian sorghum gene pool (Amare et al., 2015; Girma et al., 2019; Enyew et al., 2021), which has been used as a source for resistance to biotic (Wu et al., 2006) and abiotic traits such as stay-green (Borrell et al., 2000). Genotype B35, derived from a cross between two Ethiopian durra sorghum genotypes (BT×642), is among the well-known drought-tolerant genotypes and has been used as a major source of stay-green genes in sorghum breeding programs (Evans et al., 2013; Abreha et al., 2022). Whereas, Melkam, Argiti, and ESH4 are drought tolerant and high-yielding improved varieties being widely cultivated in Ethiopia. In the present study, genotypes G48, G65, and G66 for CHLF, G59 for CHLM and G22 and G66 for SG had higher mean performance than B35 as well as the improved varieties (Melkam, Argiti, and ESH4). Interestingly, these genotypes with a high mean performance for CHLF, CHLM and SG had high mean grain yield and other farmer preferred traits, such as plant height as reported in our previous work (Enyew et al., 2021). Hence, this study suggests the potential to discover sorghum genotypes with a stronger drought-tolerance within the Ethiopian sorghum gene pool. Further study that includes these desirable genotypes is required to gain deeper insight into the genetic control mechanisms of these drought-tolerance related traits. These results further highlight the potential of germplasm maintained in gene banks in contributing new genetic sources required for crop improvement.

In AMMI analysis, low values of the stability parameters, ASV, EV, SIPC, WAAS and ZA indicate high stability of genotypes and low G×E interaction (Sneller et al., 1997; Purchase et al., 2000; Zali et al., 2012; Pour-Aboughadareh et al., 2022). In this study, G244 was identified as the most stable genotype across the three locations based on all AMMI stability parameters, followed by G222 and G201, which were identified based on their ASV, EV and SIPC values for chlorophyll content at the flowering stage. For chlorophyll content at the maturity stage, G114, G302, and G240 showed high stability according to their EV, SIPC, WAAS and ZA values while ASV suggests that G81, G53 and G255 were the most stable. Among the top three stable genotypes identified by all AMMI stability parameters, G53 and G114 had the highest CHLF value while G201 and G222 had the highest CHLM value. All other genotypes had CHLF and CHLM values lower than the corresponding mean values. However, stable genotypes could have low mean performance, and so they should not be the only factors to select germplasm for breeding. In the present study, the top ranking genotypes which had both high mean performance and stability were selected based on their GSI, which is calculated based on ASV and genotype mean ranking (Farshadfar, 2008; Mahmodi et al., 2011). Accordingly, G222, G53 and G239 for CHLF and G53, G308 and G54 for CHLM were identified as stable genotypes across environments with high mean performance. These genotypes were originally collected from Tigray and Oromia regions, suggesting the significance of these areas as potential sources for drought tolerant genotypes. Among the stable genotypes with high mean performance, G54 and G239 had high grain yield, as shown in our previous work (Enyew et al., 2021). Therefore, these genotypes could be prioritized for use in sorghum breeding programs to enhance the desirable characteristics of these drought-tolerance related traits with the ultimate goal of developing drought tolerant cultivars. This GSI method has been effectively applied to select the top ranking and stable genotypes in sorghum and other crops (Mohammadi et al., 2010; Nduwumuremyi et al., 2017; Donkor et al., 2020; Enyew et al., 2021).

The best performing genotypes could be selected through interpreting the G×E interaction, MGE clustering, and Which-Won-Where GGE biplot that show a particular adaptation of genotypes (Gauch, 1992; Yan et al., 2000; Yan and Tinker, 2006; Rakshit et al., 2012). In the GGE biplot, the vertex genotypes located away from the biplot origin are more reactive to environmental changes and considered as specifically adapted genotypes (Yan and Kang, 2002). These genotypes could be least or top performing in some or in all tested environments (Yan and Kang, 2002). According to the ‘Which-Won-Where’ GGE biplot, the testing environments were clustered into two MGEs in this study with different top performing genotypes for CHLF and CHLM. For both CHLF and CHLM, MGE1 was represented by MH and MK environments, and contained G48 and G65 for CHLF, and G316 and G59 for CHLM as top performing genotypes. Whereas, MGE2 comprised only environment MS where G117 and G175 for CHLF and G48 for CHLF had the top mean values. The results suggest the genotypes have different levels of adaptation to different MGEs, signifying the role of G×E interactions (Mohammadi et al., 2010). Several authors have reported the identification of top performing genotypes adapted to a specific MGE (Rakshit et al., 2012; Aruna et al., 2016; Yihunie and Gesesse, 2018; Vaezi et al., 2019).

GGE ranking biplot are commonly used to select top ranking and stable genotypes across environments through based on AEC (Yan, 2001). In this study, the AEC in ranking biplot showed genotypes G48, G66, G65 and G175 as the top ranking in CHLF and G59, G321, G48, G114 and G18 for CHLM. However, due to the G×E interaction effect, the top ranking genotypes such as G59 for CHLF and G59 and G48 for CHLM were less stable. One of the major applications of the ranking biplot is to detect genotypes with both high mean performance and high stability. In this study, GGE ranking biplot identified G48, G66 and G175 for CHLF and G118, G114 and G321 for CHLM as high performing stable genotypes. The identified stable sorghum genotypes that had the highest mean performance with respect to their chlorophyll content could be used as potential novel sources of drought tolerance for sorghum improvement. It indicates that selection of best genotypes through ranking biplot analysis is a valuable approach for identifying high performing stable genotypes as shown in previous studies (Aruna et al., 2016; Oliveira et al., 2020).

## 5 Conclusion

Phenotypic evaluation is vital for efficient selection of crop germplasm for conservation and use in plant breeding programs. In this regard, the landrace accessions remain a vital source of novel alleles for traits of interest. Ethiopian sorghum landrace accessions possess significant phenotypic variation for all drought-tolerance related traits (SG, CHLM, CHLF, GLNF, FLA, PE, and PD) targeted in the present study. This suggests the high possibility of identifying novel drought-tolerant sorghum genotypes within the Ethiopian sorghum gene pool, which consists of a large collection of sorghum germplasm in the national gene bank, which has not yet been evaluated for drought tolerance. High broad-sense heritability observed for the studied traits suggests a strong genetic control, which is a favorable opportunity for improving the traits through crossbreeding and selection. The analysis of variance confirmed that genotype, environment, and G×E interaction have significant effects on the studied traits. Hence, multi-environment field trials should be conducted for reliable identification of top performing and stable genotypes in terms of drought tolerance. Overall, G222, G53, G239, G48, G66, and G175 for CHLF, and G53, G308, G54, G118, G114, and G321 for CHLM were identified as stable genotypes with high mean performance, and could be prioritized for use in sorghum breeding programs. The landrace accessions identified as having a better mean performance than the improved varieties with respect to their chlorophyll content could be potential sources of novel alleles for drought tolerance. Hence, they should be further investigated through methods such as genome-wide association studies.

## Data availability statement

The original contributions presented in the study are included in the article/**Supplementary Material**. Further inquiries can be directed to the corresponding author.

## Author contributions

All authors conceived and designed the experiment. ME conducted the experiment, analyzed the data and wrote the draft manuscript. MG, TF, AC, CH, KT and AS reviewed the manuscript. All authors contributed to the article and approved the submitted version.

## References

[B1] Abreha K. B. Enyew M. Carlsson A. S. Vetukuri R. R. Feyissa T. Motlhaodi T. . (2022). Sorghum in dryland: morphological, physiological, and molecular responses of sorghum under drought stress. Planta 255, 1–23. doi: 10.1007/s00425-021-03799-7 PMC8665920 34894286

[B2] Adugna A. (2014). Analysis of *in situ* diversity and population structure in Ethiopian cultivated *Sorghum bicolor* (L.) landraces using phenotypic traits and SSR markers. SpringerPlus 3, 1–14. doi: 10.1186/2193-1801-3-212 24877027 PMC4033718

[B3] Adugna A. Snow A. A. Sweeney P. M. Bekele E. Mutegi E. (2013). Population genetic structure of *in situ* wild *Sorghum bicolor* in its Ethiopian center of origin based on SSR markers. Genet. Resour. Crop Evol. 60, 1313–1328. doi: 10.1007/s10722-012-9921-8

[B4] Alvarado G. Rodríguez F. M. Pacheco A. Burgueño J. Crossa J. Vargas M. . (2020). META-r: A software to analyze data from multi-environment plant breeding trials. Crop J. 8, 745–756. doi: 10.1016/j.cj.2020.03.010

[B5] Amare K. Zeleke H. Bultosa G. (2015). Variability for yield, yield related traits and association among traits of sorghum (*Sorghum bicolor* (L.) moench) varieties in wollo, Ethiopia. J. Plant Breed. Crop Sci. 7, 125–133. doi: 10.5897/JPBCS2014.0469

[B6] Aruna C. Rakshit S. Shrotria P. Pahuja S. Jain S. Siva Kumar S. . (2016). Assessing genotype-by-environment interactions and trait associations in forage sorghum using GGE biplot analysis. J. Agric. Sci. 154, 73–86. doi: 10.1017/S0021859615000106

[B7] Assefa Y. Staggenborg S. A. Prasad V. P. (2010). Grain sorghum water requirement and responses to drought stress: A review. Crop Manage. 9, 1–11. doi: 10.1094/CM-2010-1109-01-RV

[B8] Badran A. (2020). Genetic parameters of some sorghum (*Sorghum bicolor* (L.)) genotypes under water deficit stress. Egypt J. Desert Res. 70, 103–119. doi: 10.21608/ejdr.2020.25119.1071

[B9] Bobade P. Amarshettiwar S. Rathod T. Ghorade R. Kayande N. Yadav Y. (2019). Effect of polyethylene glycol induced water stress on germination and seedling development of rabi sorghum genotypes. J. pharmacogn phytochem 8, 852–856.

[B10] Borrell A. K. Hammer G. L. Douglas A. C. (2000). Does maintaining green leaf area in sorghum improve yield under drought? i. leaf growth and senescence. Crop Sci. 40, 1026–1037. doi: 10.2135/cropsci2000.4041026x

[B11] de Mendiburu F. de Mendiburu M. F. (2019). Package a’gricolae’ (R Package) 1, 3–5.

[B12] Dillon S. L. Shapter F. M. Henry R. J. Cordeiro G. Izquierdo L. Lee L. S. (2007). Domestication to crop improvement: genetic resources for sorghum and s accharum (*Andropogoneae*). Ann. Bot. 100, 975–989. doi: 10.1093/aob/mcm192 17766842 PMC2759214

[B13] Disasa T. Feyissa T. Admassu B. Paliwal R. De Villiers S. M. Odeny D. A. (2016). Molecular evaluation of Ethiopian sweet sorghum germplasm and their contribution to regional breeding programs. Aust. J. Crop Sci. 10: 520–527. doi: 10.21475/ajcs.2016.10.04.p7286x

[B14] Djanaguiraman M. Prasad P. Ciampitti I. Talwar H. S. (2020). “Impacts of abiotic stresses on sorghum physiology,” in Sorghum in the 21st century: Food–Fodder–Feed–Fuel for a rapidly changing world (Singapore: Springer), 157–188.

[B15] Doggett H. (1970). Sorghum. (London, England: Longman, Tropical Agriculture Series).

[B16] Donkor E. F. Nyadanu D. Akromah R. Osei K. (2020). Genotype-by-environment interaction and stability of taro [Colocasia esculenta (l.) schott.] genotypes for yield and yield components. Ecol. Genet. Genom. 17, 100070. doi: 10.1016/j.egg.2020.100070

[B17] Dykes L. (2019). Sorghum phytochemicals and their potential impact on human health. Methods Mol. Biol. 1931, 121–140. doi: 10.1007/978-1-4939-9039-9_9 30652287

[B18] Ejeta G. (2005). “Integrating biotechnology, breeding, and agronomy in the control of the parasitic weed striga spp in sorghum,” in The wake of the double helix: from the green revolution to the gene revolution, Bologna Bologna. Eds. Tuberosa R. Phillips R. L. Gale M. . (Bologna: Avenue Media) 239–251.

[B19] Enyew M. Feyissa T. Carlsson A. S. Tesfaye K. Hammenhag C. Geleta M. (2022). Genetic diversity and population structure of sorghum [*Sorghum bicolor* (L.) moench] accessions as revealed by single nucleotide polymorphism markers. Front. Plant Sci. 12. doi: 10.3389/fpls.2021.799482 PMC8766336 35069657

[B20] Enyew M. Feyissa T. Geleta M. Tesfaye K. Hammenhag C. Carlsson A. S. (2021). Genotype by environment interaction, correlation, AMMI, GGE biplot and cluster analysis for grain yield and other agronomic traits in sorghum (*Sorghum bicolor* l. moench). PloS One 16, e0258211. doi: 10.1371/journal.pone.0258211 34610051 PMC8491923

[B21] Evans J. Mccormick R. F. Morishige D. Olson S. N. Weers B. Hilley J. . (2013). Extensive variation in the density and distribution of DNA polymorphism in sorghum genomes. PloS One 8, e79192. doi: 10.1371/journal.pone.0079192 24265758 PMC3827139

[B22] Faostat (2020). Food and agriculture organization of the united nations. Rome, Lazio, Italy: FAO. Available at: https://www.fao.org/faostat/en/#data/QCL.

[B23] Farshadfar E. (2008). Incorporation of AMMI stability value and grain yield in a single non-parametric index (GSI) in bread wheat. Pak. J. Biol. Sci. 11, 1791. doi: 10.3923/pjbs.2008.1791.1796 18817218

[B24] Gauch J. H. (1992). Statistical analysis of regional yield trials: AMMI analysis of factorial designs (Amsterdam: Elsevier Science Publishers).

[B25] Geremew G. Adugna A. Taye T. Tesfaye T. Ketema B. Michael H. (2004). Development of sorghum varieties and hybrids for dryland areas of Ethiopia. Uganda J. Agric. Sci. 9, 594–605. doi: 10.5897/AJAR2020.14801

[B26] Girma G. Nida H. Seyoum A. Mekonen M. Nega A. Lule D. . (2019). A large-scale genome-wide association analyses of Ethiopian sorghum landrace collection reveal loci associated with important traits. Front. Plant Sci. 10, 691. doi: 10.3389/fpls.2019.00691 31191590 PMC6549537

[B27] Hadebe S. Modi A. Mabhaudhi T. (2017). Drought tolerance and water use of cereal crops: A focus on sorghum as a food security crop in sub-Saharan Africa. J. Agron. Crop Sci. 203, 177–191. doi: 10.1111/jac.12191

[B28] Harris K. Subudhi P. Borrell A. Jordan D. Rosenow D. Nguyen H. . (2007). Sorghum stay-green QTL individually reduce post-flowering drought-induced leaf senescence. J. Exp. Bot. 58, 327–338. doi: 10.1093/jxb/erl225 17175550

[B29] Haussmann B. Mahalakshmi V. Reddy B. Seetharama N. Hash C. Geiger H. (2002). QTL mapping of stay-green in two sorghum recombinant inbred populations. Theor. Appl. Genet. 106, 133–142. doi: 10.1007/s00122-002-1012-3 12582881

[B30] Hou X. Xue Q. Jessup K. E. Zhang Y. Blaser B. Stewart B. . (2021). Effect of nitrogen supply on stay-green sorghum in differing post-flowering water regimes. Planta 254, 1–11. doi: 10.1007/s00425-021-03712-2 34477992

[B31] International, V (2009). Genstat for windows (Hemel Hempstead, UK: VSN Int).

[B32] Kamal N. M. Gorafi Y. S. A. Abdelrahman M. Abdellatef E. Tsujimoto H. (2019). Stay-green trait: A prospective approach for yield potential, and drought and heat stress adaptation in globally important cereals. Int. J. Mol. Sci. 20, 5837. doi: 10.3390/ijms20235837 31757070 PMC6928793

[B33] Kapanigowda M. H. Perumal R. Djanaguiraman M. Aiken R. M. Tesso T. Prasad P. V. . (2013). Genotypic variation in sorghum [*Sorghum bicolor* (L.) moench] exotic germplasm collections for drought and disease tolerance. SpringerPlus 2, 1–13. doi: 10.1186/2193-1801-2-650 24349954 PMC3863401

[B34] Kebede H. Subudhi P. Rosenow D. Nguyen H. (2001). Quantitative trait loci influencing drought tolerance in grain sorghum (*Sorghum bicolor* l. moench). Theor. Appl. Genet. 103, 266–276. doi: 10.1007/s001220100541

[B35] Kyratzis A. C. Nikoloudakis N. Katsiotis A. (2019). Genetic variability in landraces populations and the risk to lose genetic variation. the example of landrace ‘Kyperounda’and its implications for ex situ conservation. PloS One 14, e0224255. doi: 10.1371/journal.pone.0224255 31661501 PMC6818954

[B36] Liang Y. Gao Q. Xue X. (2013). Grey correlation analysis of harvest index and agronomic traits in wheat. J. Biomath 28, 335–360. doi: 10.1007/s10722-006-9123-3

[B37] Mahmodi N. Yaghotipoor A. Farshadfar E. (2011). AMMI stability value and simultaneous estimation of yield and yield stability in bread wheat ('*Triticum aestivum*' l.). Aust. J. Crop Sci. 5, 1837. doi: 10.3316/informit.005709931410019

[B38] Maluk M. D. (2018). Assessment of drought tolerance, earliness and grain yield in south Sudan sorghum germplasm and icrisat lines (Nairobi: University of Nairobi).

[B39] Mohammadi R. Haghparast R. Amri A. Ceccarelli S. (2010). Yield stability of rainfed durum wheat and GGE biplot analysis of multi-environment trials. Crop Pasture Sci. 61, 92–101. doi: 10.1071/CP09151

[B40] Mutava R. Prasad P. Tuinstra M. Kofoid K. Yu J. (2011). Characterization of sorghum genotypes for traits related to drought tolerance. Field Crops Res. 123, 10–18. doi: 10.1016/j.fcr.2011.04.006

[B41] Naoura G. Sawadogo N. Atchozou E. A. Emendack Y. Hassan M. A. Reoungal D. . (2019). Assessment of agro-morphological variability of dry-season sorghum cultivars in Chad as novel sources of drought tolerance. Sci. Rep. 9, 1–12. doi: 10.1038/s41598-019-56192-6 31863053 PMC6925278

[B42] Nduwumuremyi A. Melis R. Shanahan P. Theodore A. (2017). Interaction of genotype and environment effects on important traits of cassava (*Manihot esculenta crantz*). Crop J. 5, 373–386. doi: 10.1016/j.cj.2017.02.004

[B43] Ochieng G. Ngugi K. Wamalwa L. Manyasa E. Muchira N. Nyamongo D. . (2020). Novel sources of drought tolerance from landraces and wild sorghum relatives. Crop Sci. (TSI) 61, 104–118. doi: 10.1002/csc2.20300

[B44] Oliveira I. C. M. Guilhen J. H. S. De Oliveira Ribeiro P. C. Gezan S. A. Schaffert R. E. Simeone M. L. F. . (2020). Genotype-by-environment interaction and yield stability analysis of biomass sorghum hybrids using factor analytic models and environmental covariates. Field Crops Res. 257, 107929. doi: 10.1016/j.fcr.2020.107929

[B45] Olivoto T. Lúcio A. D. C. (2020). Metan: An r package for multi-environment trial analysis. Methods Ecol. Evol. 11, 783–789. doi: 10.1111/2041-210X.13384

[B46] Olivoto T. Lúcio A. D. Da Silva J. A. Marchioro V. S. De Souza V. Q. Jost E. (2019). Mean performance and stability in multi-environment trials I: combining features of AMMI and BLUP techniques. J. Agron. 111, 2949–2960. doi: 10.2134/agronj2019.03.0220

[B47] Peterson B. G. Carl P. Boudt K. Bennett R. Ulrich J. Zivot E. . (2014). PerformanceAnalytics: Econometric tools for performance and risk analysis. R Package version 1.

[B48] Pour-Aboughadareh A. Khalili M. Poczai P. Olivoto T. (2022). Stability indices to deciphering the genotype-by-Environment interaction (GEI) effect: An applicable review for use in plant breeding programs. Plants 11, 414. doi: 10.3390/plants11030414 35161396 PMC8839246

[B49] Praba M. L. Cairns J. Babu R. Lafitte H. (2009). Identification of physiological traits underlying cultivar differences in drought tolerance in rice and wheat. J. Agron. Crop Sci. 195, 30–46. doi: 10.1111/j.1439-037X.2008.00341.x

[B50] Purchase J. Hatting H. Van Deventer C. (2000). Genotype× environment interaction of winter wheat (Triticum aestivum l.) in south Africa: II. stability analysis of yield performance. Afr J. Plant Soil 17, 101–107. doi: 10.1080/02571862.2000.10634878

[B51] Queiroz M. S. Oliveira C. E. Steiner F. Zuffo A. M. Zoz T. Vendruscolo E. P. . (2019). Drought stresses on seed germination and early growth of maize and sorghum. J. Agric. Sci. 11, 310–318. doi: 10.5539/jas.v11n2p310

[B52] Rakshit S. Ganapathy K. Gomashe S. Rathore A. Ghorade R. Kumar M. N. . (2012). GGE biplot analysis to evaluate genotype, environment and their interactions in sorghum multi-location data. Euphytica 185, 465–479. doi: 10.1007/s10681-012-0648-6

[B53] Reddy N. R. R. Ragimasalawada M. Sabbavarapu M. M. Nadoor S. Patil J. V. (2014). Detection and validation of stay-green QTL in post-rainy sorghum involving widely adapted cultivar, M35-1 and a popular stay-green genotype B35. BMC Genomics 15, 1–16. doi: 10.1186/1471-2164-15-909 25326366 PMC4219115

[B54] Reddy B. V. Ramaiah B. Ashok Kumar A. Reddy P. S. (2007). Evaluation of sorghum genotypes for the stay-green trait and grain yield. J. SAT Agric. Res. 3, 1–4.

[B55] Sneller C. Kilgore-Norquest L. Dombek D. (1997). Repeatability of yield stability statistics in soybean. Crop Sci. 37, 383–390. doi: 10.2135/cropsci1997.0011183X003700020013x

[B56] Stamenković O. S. Siliveru K. Veljković V. B. Banković-Ilić I. B. Tasić M. B. Ciampitti I. A. . (2020). Production of biofuels from sorghum. Renew Sustain. 124, 109769. doi: 10.1016/j.rser.2020.109769

[B57] Stickler F. Wearden S. Pauli A. (1961). Leaf area determination in grain sorghum 1. J. Agron. 53, 187–188. doi: 10.2134/agronj1961.00021962005300030018x

[B58] Sukumaran S. Li X. Li X. Zhu C. Bai G. Perumal R. . (2016). QTL mapping for grain yield, flowering time, and stay-green traits in sorghum with genotyping-by-sequencing markers. Crop Sci. 56, 1429–1442. doi: 10.2135/cropsci2015.02.0097

[B59] Tao Y. Henzell R. Jordan D. Butler D. Kelly A. Mcintyre C. (2000). Identification of genomic regions associated with stay green in sorghum by testing RILs in multiple environments. Theor. Appl. Genet. 100, 1225–1232. doi: 10.1007/s001220051428

[B60] Team, R.C ., (2020). R: A language and environment for statistical computing (Vienna, Austria: R Foundation for Statistical Computing). Available at: https://www.R-project.org/.

[B61] Teressa T. Bejiga T. Semahegn Z. Seyoum A. Kinfe H. Nega A. . (2021). Evaluation of advanced sorghum (*Sorghum bicolor* l. moench) hybrid genotypes for grain yield in moisture stressed areas of Ethiopia. Int. J. Agric. Sci. Food Technol. 7, 212–219. doi: 10.17352/2455-815X.000109

[B62] Thomas H. Ougham H. (2014). The stay-green trait. Theor. Appl. Genet. 65, 3889–3900. doi: 10.1093/jxb/eru037 24600017

[B63] Tsuda M. (1986). Effects of water deficits on panicle exsertion in rice (Oryza sativa l.) and sorghum (*Sorghum bicolor* (L.) moench). Jpn. J. Crop Sci. 55, 196–200. doi: 10.1626/jcs.55.196

[B64] Tuinstra M. Grote E. Goldsbrough P. Ejeta G. (1996). Identification of quantitative trait loci associated with pre-flowering drought tolerance in sorghum. Crop Sci. 36, 1337–1344. doi: 10.2135/cropsci1996.0011183X003600050043x

[B65] Vaezi B. Pour-Aboughadareh A. Mohammadi R. Mehraban A. Hossein-Pour T. Koohkan E. . (2019). Integrating different stability models to investigate genotype× environment interactions and identify stable and high-yielding barley genotypes. Euphytica 215, 63. doi: 10.1007/s10681-019-2386-5

[B66] Wagaw K. Seyoum A. Tadesse T. Gebreyohannes A. Nega A. Tadesse D. (2020). The MET analysis of yield performance of advanced sorghum [*Sorghum bicolor* (L.) moench] lines under moisture stress areas using spatial analysis. Am. J. Plant Sci. 11, 1618. doi: 10.4236/ajps.2020.1110117

[B67] Waniska R. Rooney L. Mcdonough C. (2016). "Sorghum: utilization", in Encyclopedia of food grains, the world of food grains. Eds. Wrigley C. Corke H. Seetharaman K. Faubion J. . (Oxford: Elsevier) 116– 123.

[B68] Wanous M. K. Miller F. R. Rosenow D. T. (1991). Evaluation of visual rating scales for green leaf retention in sorghum. Crop Sci. 31, 1691–1694. doi: 10.2135/cropsci1991.0011183X003100060063x

[B69] Wondimu Z. Bantte K. Paterson A. H. Worku W. (2020). Agro-morphological diversity of Ethiopian sorghum [*Sorghum bicolor* (L.) moench] landraces under water limited environments. Genet. Resour. Crop Evol. 67, 2149–2160. doi: 10.1007/s10722-020-00968-7

[B70] Wu Y. Huang Y. Tauer C. Porter D. R. (2006). Genetic diversity of sorghum accessions resistant to greenbugs as assessed with AFLP markers. Genome 49, 143–149. doi: 10.1139/g05-095 16498464

[B71] Xu W. Subudhi P. K. Crasta O. R. Rosenow D. T. Mullet J. E. Nguyen H. T. (2000). Molecular mapping of QTLs conferring stay-green in grain sorghum (*Sorghum bicolor* l. moench). Genome 43, 461–469. doi: 10.1139/g00-003 10902709

[B72] Yan W. (2001). GGEbiplot–a windows application for graphical analysis of multienvironment trial data and other types of two-way data. Crop Sci. 93, 1111–1118. doi: 10.2134/agronj2001.9351111x

[B73] Yan W. Hunt L. Sheng Q. Szlavnics Z. (2000). Cultivar evaluation and mega-environment investigation based on the GGE biplot. Crop Sci. 40, 597–605. doi: 10.2135/cropsci2000.403597x

[B74] Yan W. Kang M. S. (2002). GGE biplot analysis: A graphical tool for breeders, geneticists, and agronomists (Boca Raton: CRC press).

[B75] Yan W. Tinker N. A. (2005). An integrated biplot analysis system for displaying, interpreting, and exploring genotype× environment interaction. Crop Sci. 45, 1004–1016. doi: 10.2135/cropsci2004.0076

[B76] Yan W. Tinker N. A. (2006). Biplot analysis of multi-environment trial data: Principles and applications. Can. J. Plant Sci. 86, 623–645. doi: 10.4141/P05-169

[B77] Yihunie T. A. Gesesse C. A. (2018). GGE biplot analysis of genotype by environment interaction in field pea (*Pisum sativum* l.) genotypes in northwestern Ethiopia. J. Crop Sci. Biotechnol. 21, 67–74. doi: 10.1007/s12892-017-0099-0

[B78] Zali H. Farshadfar E. Sabaghpour S. H. Karimizadeh R. (2012). Evaluation of genotype× environment interaction in chickpea using measures of stability from AMMI model. Ann. Biol. Res. 3, 3126–3136.

