# Supplementary material for: Novel sources of drought tolerance in sorghum landraces revealed via the analyses of genotype-by-environment interactions
Source: Front Plant Sci. 2022 Dec 7;13:1062984. doi: 10.3389/fpls.2022.1062984 (PMC9768483; doi:10.3389/fpls.2022.1062984)
Supplement: Supplementary file 3 [file Table_3.docx]

**Supplementary Table 3.** Combined analysis of variance for drought-tolerance related traits of 324 sorghum genotypes across three environments.

|  |  | **CHLF** |  | **CHLM** |  | **GLNF** |  | **SG** |  | **FLA** |  | **PAE** |  | **PDL** |  |
| --- | --- | --- | --- | --- | --- | --- | --- | --- | --- | --- | --- | --- | --- | --- | --- |
|  | DF | SS | MS | SS | MS | SS | MS | SS | MS | SS | MS | SS | MS | SS | MS |
| **GEN** | 323 | 38718 | 119.9*** | 26976.1 | 83.5*** | 2881.26 | 8.92*** | 537.76 | 1.67*** | 2419470.0 | 7491.0*** | 27695.1 | 85.74*** | 87171.0 | 269.9*** |
| **ENV** | 2 | 9654 | 4826.9*** | 2812.4 | 1406.2*** | 42.08 | 21.04*** | 226.14 | 113.07*** | 4942146.0 | 2471073.0*** | 3609.6 | 1804.81*** | 3854.0 | 1926.8*** |
| **REP:ENV** | 3 | 604 | 201.4*** | 528 | 176.0*** | 6.70 | 2.23 | 27.64 | 9.21*** | 19367.0 | 6456.0* | 409.6 | 136.53** | 17354.0 | 5784.6*** |
| **G×E** | 646 | 37071 | 57.4*** | 20926.2 | 32.4*** | 1562.06 | 2.42* | 359.04 | 0.56 | 1764381.0 | 2731.0** | 30853.7 | 47.76*** | 65124.0 | 100.8*** |
| **BLK:ENV:REP** | 66 | 3550 | 53.8** | 2684.5 | 40.7** | 191.19 | 2.90* | 59.08 | 0.90** | 204482.0 | 3098.0* | 2332.5 | 35.34 | 9522.0 | 144.3*** |
| **Residuals** | 903 | 30544 | 33.8 | 22613.1 | 25.04 | 1894.63 | 2.10 | 495.61 | 0.55 | 1989171.0 | 2203.0 | 27814.7 | 30.8 | 52086.0 | 57.7 |

*** = Significant at 0.001 significance level; ** = Significant at 0.01 significance level; * = Significant at 0.05 significance level; DF = Degrees of freedom; GEN = Genotype; REP = Replication; ENV = Environment; BLK = Block; SS = Sum of squares; MS = Mean square, CHLF = Chlorophyll content at flowering; CHLM = Chlorophyll content at maturity; SG = Stay-green; GLNF = Green leaf number at flowering; FLA = Flag leaf area; PAE = Panicle exertion; PDL = Peduncle length.
